# Analysis of postoperative infection factors of retrograde intrarenal surgery combined with negative pressure equipment for renal stones

**DOI:** 10.1038/s41598-024-75073-1

**Published:** 2024-10-03

**Authors:** Deheng Cui, Qinghong Ma, Qiuyan Zhang, Lian Zhang, Guoqiang Chen

**Affiliations:** Department of Urology, The Second Hospital of Longyan, Longyan, 364000 Fujian China

**Keywords:** Negative pressure, Retrograde intrarenal surgery, Sepsis, Infection factors, Renal calculi, Urinary tract infection

## Abstract

Our study aimed to retrospectively analyze the patients who underwent the combination of negative pressure equipment and RIRS(NP-RIRS) for renal stones, and identified new high-risk factors related to infection. 456 patients with renal stones ≤ 3 cm underwent NP-RIRS in our department, from January 2022 to October 2023. All patients were divided into non-infection group(NIRIRS group) and infection group(IRIRS group) based on infection complications during the perioperative period. Establish a receiver operating characteristic curve(ROC curve)based on variables with statistical differences, and use the Jordan index to find the optimal cutoff value. Classify the data into two categories based on the best truncation value, and perform binary logistic regression analysis on the classified data. In the IRIRS group, there were 10 cases of fever (2.92%), 2 cases of sepsis (0.58%), and no cases of septic shock or death. The length and the CT value of the stone were 16 (13,21) vs. 22 (19,24) (p < 0.001), 764 (570,1012) vs. 1372 (841,1527) (p < 0.001), respectively, and there was a statistical difference. The surgical time of NIRIRS group and IRIRS group were 57 (50,65) vs. 75 (60,98), respectively (p < 0.001), with statistical differences. On the contrary, the stone-free rate(SFR) at 3 months after surgery was 97.60% vs. 91.70% (p = 0.209), and there was no difference. The length of stones, surgical time, and CT values of stones between the two groups were further used to establish ROC, with ROC areas of 0.791, 0.791, and 0.816, respectively(Fig. 2). Based on the Jordan index, the optimal cutoff values were 17.5 mm (stone size), 64.5 min (surgery time), and 732.5 HU (stone CT value), respectively. Three continuous variables were transformed into binary data using the best truncation criterion, and the classified results were subjected to binary logistic regression analysis. The results showed that the three variables remained independent risk factors for postoperative infection complications. The incidence of infection after NPRIRS was lower than TRIRS for kidney stones. Length of stones, surgical time, and CT value of stones were independent risk factors for postoperative infection in NPRIRS treatment of kidney stones. Due to the small sample size, the credibility and generalizability of the conclusions were limited.

**Supplementary Information:**

The online version contains supplementary material available at 10.1038/s41598-024-75073-1.

## Introduction

Urinary calculi is a benign disease with a high incidence rate worldwide. Surgical treatment gradually tends to be minimally invasive, and more attention is paid to patients’ comfort and faster recovery of normal life. retrograde intrarenal surgery(RIRS) has been widely used and is often recommended for kidney stones smaller than 2 cm^1^. The traditional view is that during RIRS, the circulation of irrigation fluid is difficult, and the speed of stone removal is slow, which easily leads to high pressure of the renal pelvis and increased perioperative complications^2^. The overall complication rate after RIRS is 9–25%^3,4^. There is evidence suggesting a risk of post-operative urosepsis of up to 5%, and common risk factors are diabetes, female, positive preoperative urine culture, large stone burden, long operation time, etc^2,5^.

In recent years, the combination of negative pressure equipment and RIRS(NP-RIRS) for treating renal stones has been widely used in China, and its safety and effectiveness have also been verified^6^. However, the occurrence of sepsis during the perioperative period cannot be avoided entirely. Every occurrence of sepsis is a disaster for patients, and we need to be constantly vigilant. The previous high-risk factors may no longer be suitable for NP-RIRS. Therefore, our study aimed to analyze the patients who underwent NP-RIRS for renal stones retrospectively, identified new high-risk factors related to infection, and provided early warning for clinical physicians to reduce the incidence of infection after surgery.

## Method

456 patients with renal stones underwent NP-RIRS in our department, from January 2022 to October 2023. Inclusion criteria: Long diameter of stones or the sum of the long diameters of multiple stones ≤ 3 cm. Exclusion criteria: (1) Simultaneous surgery for bilateral urinary tract stones, (2) Uncontrolled urinary tract infection, (3) Transplanted kidneys, (4) Severe cardiopulmonary dysfunction, (5) Uncontrollable hemorrhagic disease. Based on the inclusion and exclusion criteria, 342 cases were ultimately included and divided into a non-infection group (NIRIRS, *n* = 330) and an infection group (IRIRS, *n* = 12) based on the occurrence of infection complications during the perioperative period. The data from two groups of patients were retrospectively analyzed, including age, gender, stone burden, renal function, previous surgical history, comorbidities, urine culture, renal anatomy, surgical-related parameters, and perioperative complications.

This study was approved by the Ethics Committee of the Second Hospital of Longyan City, Fujian Province, and informed consent was obtained from all patients. Our study was conducted by the ethical standards of the 1964 Declaration of Helsinki and its subsequent amendments.

### Preoperative preparation

Patients with positive urine cultures should be treated with sensitive antibiotics based on the results of drug sensitivity experiments until the urine culture turns negative. In addition, patients with negative urine cultures should receive a single dose of antibiotics (second-generation cephalosporins or quinolones) before surgery to prevent infection. For hypertensive patients, preoperative blood pressure should be controlled below 150/95 mmHg (1 mmHg = 0.133 kPa). In patients with diabetes, the fasting plasma glucose was controlled at 6–8 mmol/L. Each surgery was performed by the same experienced urologist specializing in stones.

## Evaluation indicators

Postoperative infection was considered as one of the following manifestations: (1) positive urine culture, (2) positive blood culture, (3) body temperature > 38.5 ℃ that excluding fever caused by other systemic infections and other factors. According to Sepsis-3 diagnostic criteria, sepsis was defined as life-threatening organ dysfunction caused by the host’s dysregulated response to infection, with a rapid increase in Quick SOFA score ≥ 2. Definition of septic shock: patients with sepsis undergo adequate treatment, but persistent hypotension persists after volume resuscitation, requiring vasoconstrictor drugs to maintain mean arterial pressure ≥ 65mmHg, and serum lactate level > 2mmol/L. All complications related to infection were recorded until one month after the operation^7^. Stone clearance was defined as a thin-layer renal CT scan 3 months after surgery showing no residual stones or stone fragments less than or equal to 2 mm. The operative time was calculated from the successful retention of negative pressure ureteral access sheath(NUAS) to the completion of double-J tube placement. The diameter of the stones referred to the longest diameter measured on the plain film in the CT scan.

## Surgical procedure

**Fig. 1:**
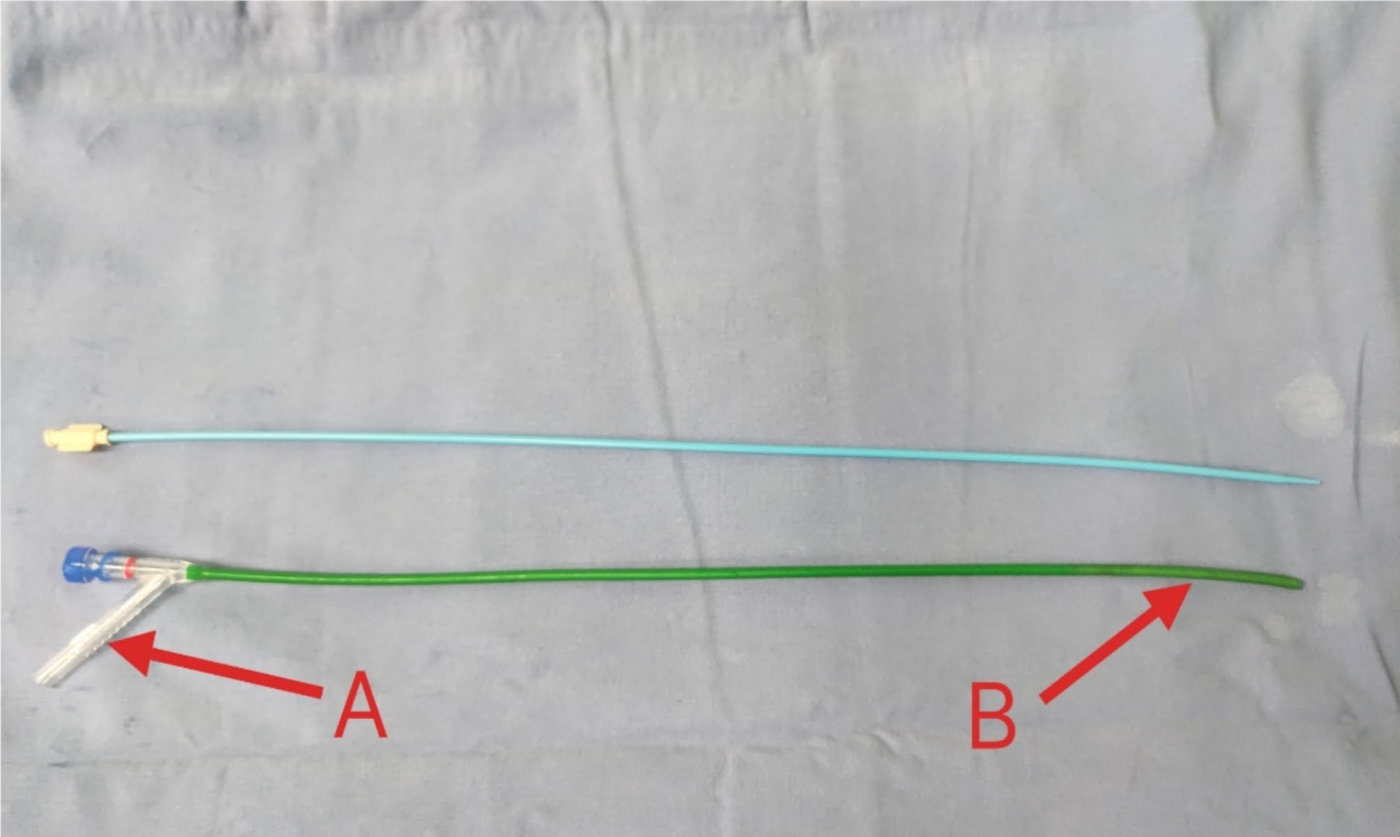
The tail end connected to negative pressure suction(A) of ureteral access sheath and the flexible head end(B).

**Fig. 2:**
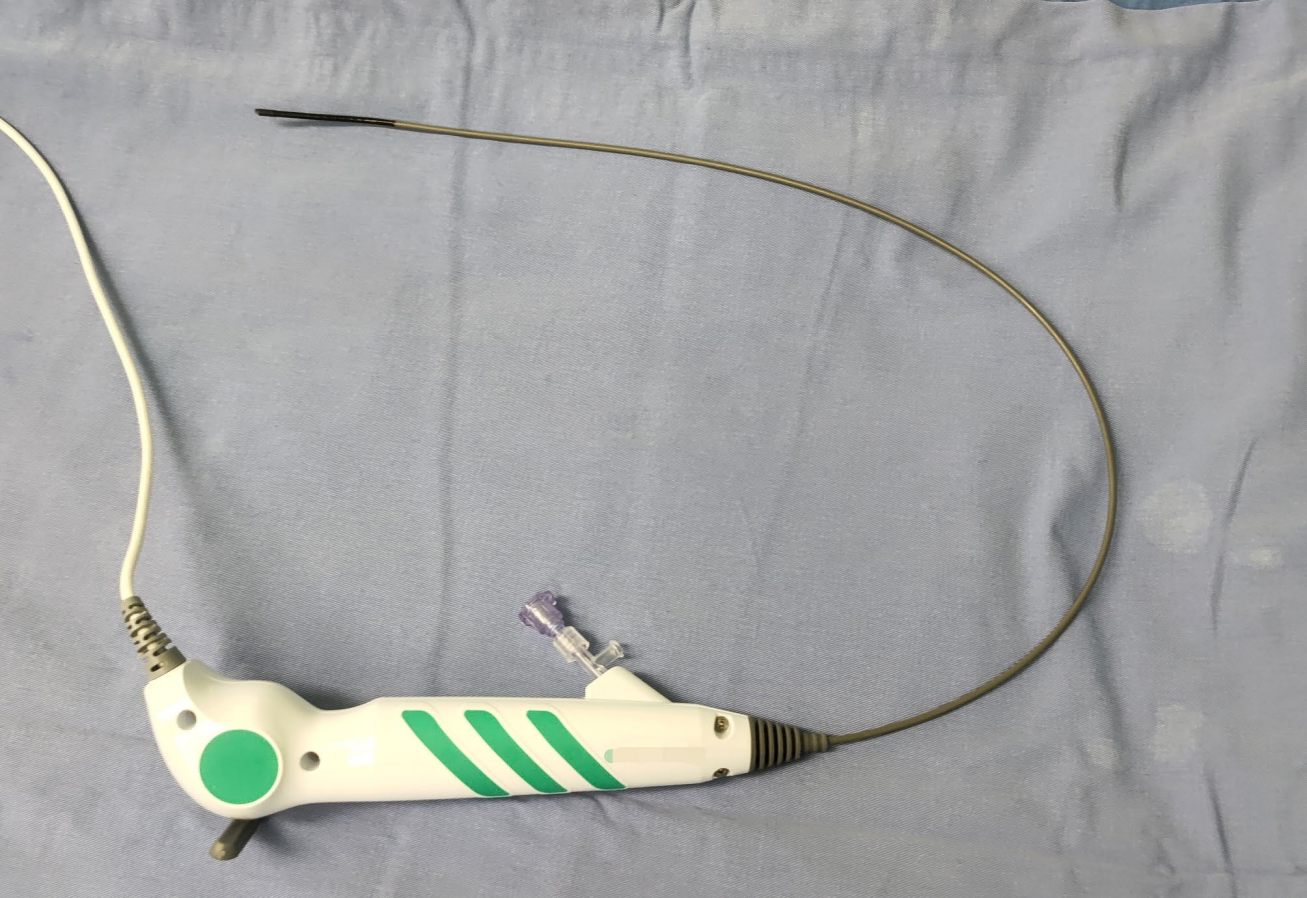
Disposable flexible ureteroscope.

All patients received general anesthesia and were in the oblique supine position. The ureter was examined by a ureteroscope and a COOK guide wire was inserted into the renal calyces. The 12 F NUAS( Fig. 1) was inserted into the renal pelvis along the guide wire. The tail of NUAS was connected to negative pressure suction, and the attraction was set to 0.02-0.04Mpa. Disposable ureteroscopy(Happiness Work, Fig. 2) combined with 200 μm holmium laser fiber (Lumenis holmium laser) was used for lithotripsy, with an energy setting of 0.6–0.8 J, frequency setting of 20–30 Hz, and perfusion flow rate of 50-150 ml/min. The flexible head of NUAS could enter almost all renal calyces and remove stone fragments quickly. A 7 F double J tube and a 16 F catheter were placed. Double J tubes were removed one month after surgery.

## Statistical method

The sample size was calculated with the formulas of logistic regression by PASS 11.0. The type-1 error (α) was set at 0.05 and the power (1 – β) at 0.8. The odds ratio was 2. The minimum sample size was 328. Quantitative data is first tested for normality and homogeneity of variance. If it follows a normal distribution and has homogeneity of variance, two independent samples for the t-test are selected, and the results are expressed as mean ± standard deviation. Suppose it does not follow a normal distribution or homogeneity of variance. In that case, the Mann-Whitney U-test is chosen, and the results are expressed as the median (upper quartile to lower quartile). Count data is represented as an example (%), grade count data is tested using Mann Whitney U test, and non-grade count data is tested using χ^2^ Inspection. *P* < 0.05 is considered statistically significant. Establish an ROC based on variables with statistical differences, and use the Jordan index to find the optimal cutoff value. Classify the data into two categories based on the best truncation value, and perform binary logistic regression analysis on the classified data. All statistical analyses were conducted using the commercially available software SPSS 27.0.

## Results

In the IRIRS group, there were 10 cases of fever (2.92%), 2 cases of sepsis (0.58%), and no septic shock or death cases. In the two groups, 26 and 2 patients, respectively, had D-J tubes placed in advance (Table 1). There was no statistically significant difference in gender, age, body mass index, ASA score, preoperative urine culture, and hydronephrosis between NIRIRS group and IRIRS group (Table 1). The NIRIRS group includes 7 horseshoe kidneys, 2 pelvic ectopic kidneys, and 5 renal malrotation. The IRIRS group has one horseshoe kidney. The distribution proportion of stones in the upper, middle, lower, renal pelvis, and multiple between the two groups was not significantly different. However, the length and the CT value of the stone were 16 (13,21) vs. 22 (19,24) (*p* < 0.001), 764 (570,1012) vs. 1372 (841,1527) (*p* < 0.001), respectively, and there was statistical difference.

**Table 1:** Comparison of preoperative demographics of patients.

Variables	Groups		p value
	Non-infectious	Infectious	
Number	330	12	-
Gender (male/female)	128/202	6/6	0.434
Age (years)	52.81 ± 11.33	52.67 ± 11.06	0.910
BMI (kg/m^2^)	23.02 ± 3.47	23.17 ± 3.57	0.883
ASA score			0.289
0	227	8	
1–2	97	3	
3–4	6	1	
DM	32	2	0.428
Urine cultures (positive/negative)	47/283	1/11	0.563
Hydronephrosis grade			0.343
No	174	8	
Mild	107	1	
Middle	33	2	
Sever	16	1	
Renal abnormality	14	1	0.497
Stone size (mm)	16(13,21)	22(19,24)	< 0.001
Stone site (left/right)	124/206	5/7	0.774
Stone location			0.190
Upper pole	30	1	
Middle pole	103	1	
Lower pole	23	2	
Pelvis	32	0	
Multiple	142	8	
Stones density (HU)	764(570,1012)	1372(841,1527)	< 0.001
Pre-op JJ stent	26	2	0.275
Previous stone treatment			0.229
SWL	28	1	
PCNL/URS	23	2	
Open surgery	10	0	

BMI body mass index, ASA score American Society of Anaesthesiologists Score, DM diabetes mellitus, HU Hounsfield Unite, URS Ureterorenoscopy, SWL shock wave lithotripsy, PCNL percutaneous nephrolithotomy.

During the surgery, there were 15 cases of bleeding, 4 cases of ureteral mucosal tear, and 3 cases of ureteral perforation in the NIRIRS group. There was 1 case of bleeding and 1 case of ureteral mucosal tear in the IRIRS group, and no ureteral perforation occurred. The surgical time of NIRIRS group and IRIRS group were 57 (50,65) vs. 75 (60,98), respectively (*p* < 0.001), with statistical differences. On the contrary, the stone-free rate (SFR) at 3 months after surgery was 97.60% vs. 91.70% (*p* = 0.209), and there was no difference (Table 2).

**Table 2:** Comparison of perioperative parameters and outcomes.

Variables	Groups		p value
	Non-infectious	Infectious	
Number	330	12	-
Surgical time (min)	57(50,65)	75(60,98)	< 0.001
Ureter dilation	17	0	0.420
NUAS size			0.131
10/12F	36	3	
12/14F	294	9	
Per-op complications			0.203
Hemorrhage	15	1	
Ureteral avulsion	4	1	
Perforation	3	0	
Stone-free status at third month (%)	97.60	91.70	0.209

NUAS negative pressure ureteral access sheath.

**Fig. 3:**
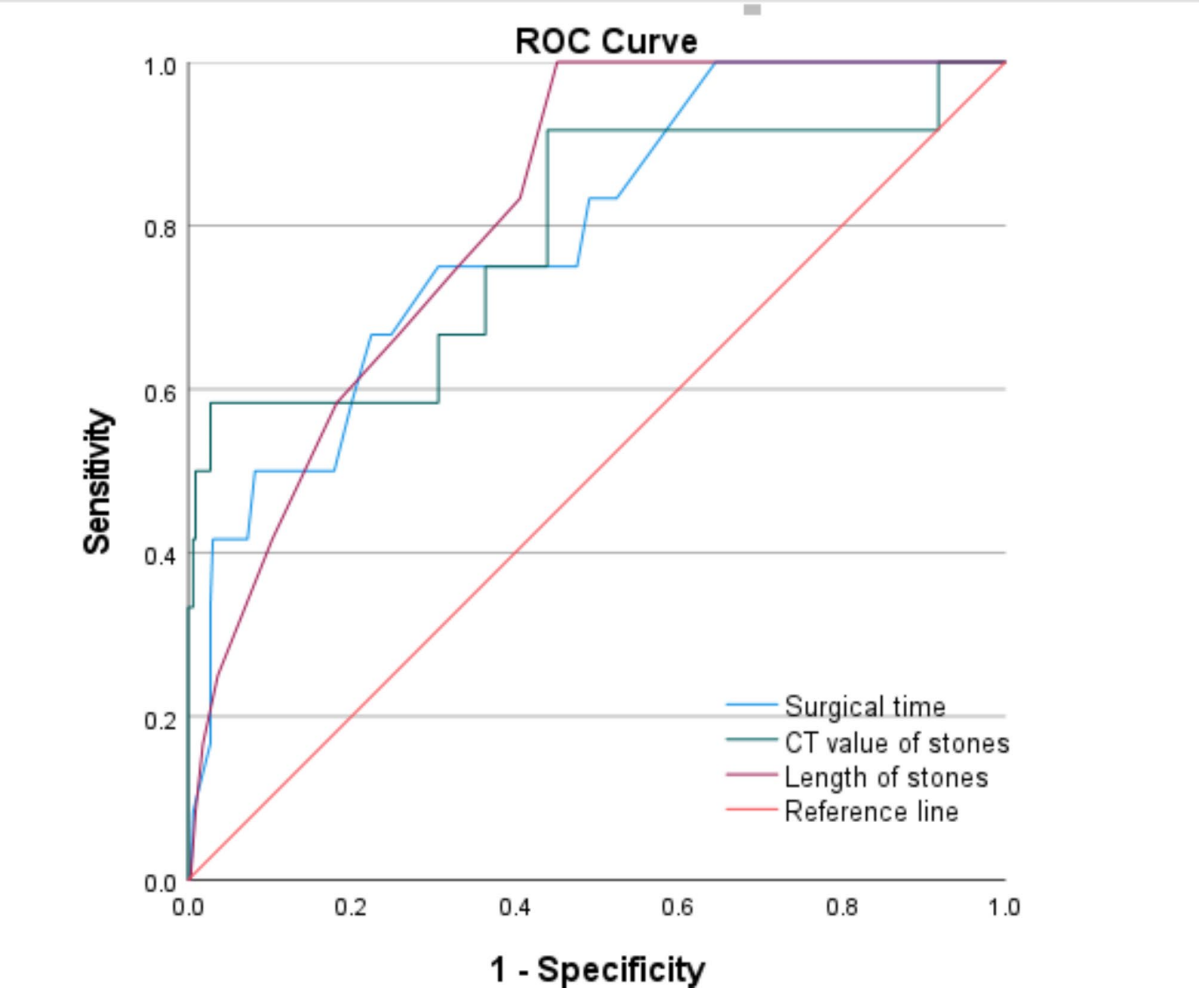
ROC curve of length of stones, surgical time, and CT values of stones.

The length of stones, surgical time, and CT values of stones between the two groups were further used to establish ROC, with ROC areas of 0.791, 0.791, and 0.816, respectively (Fig. 3). Based on the Jordan index, the optimal cutoff values were 17.5 mm (stone size), 64.5 min (surgery time), and 732.5 HU (stone CT value), respectively. Three continuous variables were transformed into binary data using the best truncation criterion, and the classified results were subjected to binary logistic regression analysis. The results showed that the three variables remained independent risk factors for postoperative infection complications (Table 3).

**Table 3:** Binary logistic regression analysis of three high-risk factors.

Variable	OR	*p* value
Surgical time	1.067	0.004
Stone size	1.785	< 0.001
Stones density	1.008	< 0.001

## Discussion

The flex ureteroscopy for renal stones has been extensively studied, and the complications have been analyzed in detail. Undoubtedly, the most harmful complication is still infection, especially sepsis, and septic shock, which may endanger the patient’s life^5^. Many studies on infection-related prevention and prediction had been conducted, which reducing positively reduced the occurrence and treatment of infections^5,8^. However, these studies mainly focused on traditional RIRS(TRIRS), and controlling renal pelvis pressure during surgery was a challenge. Our study found that the incidence of infection complications in NP-RIRS for renal stones was 2.92%, with a risk of sepsis of 0.58%, significantly lower than in TRIRS^2^. As we know, there is limited research on high-risk factors for postoperative infection in NP-RIRS. Our research findings indicated that surgical time, stone length, and stone CT value are not only high-risk factors for postoperative infection in NP-RIRS but also independent risk factors.

As is well known, there is a close correlation between surgical time and postoperative infection-related complications. Similarly, in our study, the median surgical time in the NIRIRS group was 57 min, while in the IRIRS group, it was 75 min, indicating statistically significant difference^8,9^. Ozgor et al. reviewed 463 patients who underwent RIRS and found that surgery time > 60 min was positively correlated with infection complications after RIRS^8^. Knipper et al. analyzed patient data from 2010 patients with RIRS for stones. They found that the average surgical time for patients without complications was 34 min (range 20–60 min), while for patients with complications, it was 45 min (range 25–76 min) (*p*< 0.001)^9^. Surgical time is disrupted by various factors, such as stone burden, surgical instruments, lithotripsy methods, operator experience, etc^10^. With the extension of surgical time, the possibility of the toxins released during the stone fragmentation process being absorbed was increased. However, the pressure of renal pelvis generated by lavage fluid during NP-RIRS can remain below 30mmhg, which is lower than the pressure of renal vein system and lymphatic vessels, significantly reducing the absorption of toxins^11^. Our study was limited to patients with stones less than 3 centimeters in length, with a maximum surgical time of 120 min and an optimal cutoff value of 64.5 min. Higher quality research is needed to explore whether there are different conclusions regarding larger stone lengths and longer surgical times.

TRIRS was often recommended for renal stones smaller than 2 cm due to the production of many stone fragments during surgery. Relying on stone basket to remove the stones will significantly prolong the surgical time, and patients’ self-removal of stone fragments will increase the risk of residual stones^12^. NP-RIRS is trying to change this situation. We extended indications for surgery to 3 cm and found that the optimal cutoff value for stone length was 17.5 mm. 181 (52.92%) patients had stone lengths below 17.5 mm, and no infection occurred after surgery. On the contrary, there were 161 (47.08%) patients with diameters higher than 17.5 mm, and the proportion of infection complications was as high as 7.45% (12/161). The significant increase in the incidence of the latter may be due to two factors. Firstly, large stones contained more bacteria and toxins, and the surgical time was extended accordingly. Secondly, large stones easily cause obstruction, with many bacteria breeding in closed environments. Complete obstruction leads to the inability to excrete urine in the renal pelvis, resulting in false negative urine culture and urine routine, which affects preoperative judgment of the condition^13^. Therefore, even if NPRIRS is used to treat larger renal stones, we still cannot relax our vigilance.

The CT value of stones is closely related to their density, affecting our lithotripsy method, whether to choose powdered or fragmented stones^14^. Previous studies had shown that powdered SFR is higher than fragmented SFR (65,6 vs. 87,1%, *p*= 0043), as TRIRS use auxiliary instruments such as nickel-titanium baskets to remove residual fragments, which may severely limit the movement amplitude of the flexible ureteroscopy tip^12,15^. Our negative pressure technology can directly removed stone fragments or powder, but unfortunately, our information on this aspect was not complete. In addition, we found that stones with high CT values are difficult to powder, and fragmentation is often chosen. Fragmentation might had a negative impact on the occurrence of infection-related complications. When multiple fragments accumulate inside the suction tube, it might obstructed lavage fluid reflux, slowly increased renal pelvis pressure, and be easily overlooked. At this time, if the surgeon fails to relieve the obstruction timely, very bad results might occurred because the fluid was poured into the renal pelvis by infusion pump continuously. Although our infusion pump platform does not have an intelligent alarm function, we can also detect an increase in pressure from other aspects, such as the renal pelvis becoming fuller from depression, the floating logistics in the field of view being relieved, and the speed of stone fragments flowing outward slowing down.

Elderly patients need more attention during the operation because the incidence rate of diabetes, hypertension, chronic kidney disease, and heart failure is high among the elderly. However, we found that NPRIRS for elderly patients was as safe as for young patients, and there was no significant difference in age between the two groups. It should be emphasized that gender, diabetes, positive preoperative urine culture and hydronephrosis are not high-risk factors for infection after NPRIRS, which differs from TRIRS^5,16,17^. These results may be due to the significant reduction in toxin absorption by negative pressure technology, which once again proves that reducing intrarenal pressure is the key to reducing infection complications^11,18^.

Currently, the use of negative pressure ureteral access sheath(NUSA) combined with RIRS for renal calculi treatment is rapidly expanding, achieving a lower postoperative infection rate compared to the traditional non-negative pressure USA combined with RIRS in experienced centers (Table 4)^19,20^. Our study discovered that low intrapelvic pressure reduced the infection rate, positive preoperative urine culture was not significant risk factor for infection. However, the applicability of this technique should not be indiscriminately expanded. As operation duration, stone burden, and CT value increase, the risk of infection continues to rise. Additionally, we summarized and shared operational techniques for NUAS combined with RIRS, which were not mentioned in the other two studies^19,20^. Our operational insights may facilitate the wider adoption of this technique. Finally, our findings alert physicians newly initiating this technique to choose suitable patients to receive the optimal treatment plan.

**Table 4:** Comparison of main information from three different studies.

Study	Ureteral access sheath	Ureteroscope type	Infectious complications incidence	Sample size, *n*	Independent risk factors
Hua Zhang etc.^1^	Non-negative pressure UAS	Reused flexible ureteroscope	43/602(7.14%)	602	1. Operative time 2. Stone size
Jinseok Kang etc.^2^	Non-negative pressure UAS	Reused flexible ureteroscope	14/243(5.76%)	243	1. Positive preoperative urine culture 2. Operative time 3. Ureter balloon dilation
Our study	Negative pressure UAS	Disposable flexible ureteroscope	12/340(3.51%)	342	1. Stone size 2. Operative time 3. Stone CT value

^1^https://doi.org/10.1177%2F0300060520956833.

^2^10.14777/uti.2023.18.3.93.

UAS: Ureteral access sheath.

Our research has some limitations. The study was retrospective, and there was no standardization in selecting research subjects and data collection. It was difficult to determine the composition of the stones before surgery accurately, so they were not included in the analysis. Given the recent application of the technology, we lack sufficient data on long-term complications. Despite the large total sample size of our study, there were only 12 patients in IRIRS group, which limited the credibility and generalizability of the results. Due to the limited number of patients with TRIRS in our center, achieving concurrent control was challenging. All patients’ D-J stents were routinely left for 1 month in our medical center. While, a shorter duration for the D-J tube, such as 2 weeks, is also a viable option. Further large-scale, multicenter, prospective, randomized controlled studies will be conducted to confirm our conclusions.

## Conclusion

The incidence of infection after NPRIRS was lower than TRIRS for kidney stones. Length of stones, surgical time, and CT value of stones were independent risk factors for postoperative infection in NPRIRS treatment of kidney stones. Due to the small sample size, the credibility and generalizability of the conclusions were limited.

## Electronic supplementary material

Below is the link to the electronic supplementary material.

Supplementary Material 1

## Data Availability

Data is provided within the manuscript or supplementary information files. All data generated or analysed during this study are included in this published article and its supplementary information files. Supplementary table S1 contains information on the collection methods for each sample.
